# Identification of a microRNA signature associated with risk of distant metastasis in nasopharyngeal carcinoma

**DOI:** 10.18632/oncotarget.3005

**Published:** 2015-01-23

**Authors:** Jeff P. Bruce, Angela B. Y. Hui, Wei Shi, Bayardo Perez-Ordonez, Ilan Weinreb, Wei Xu, Benjamin Haibe-Kains, Daryl M. Waggott, Paul C. Boutros, Brian O'Sullivan, John Waldron, Shao Hui Huang, Eric X. Chen, Ralph Gilbert, Fei-Fei Liu

**Affiliations:** ^1^ Princess Margaret Cancer Centre, University Health Network, Toronto, ON, Canada; ^2^ Department of Medical Biophysics, University of Toronto, Toronto, ON, Canada; ^3^ Department of Medicine, Stanford University, Stanford, CA, United States; ^4^ Department of Pathology, Princess Margaret Cancer Centre, University Health Network, Toronto, ON, Canada; ^5^ Division of Biostatistics, Princess Margaret Cancer Centre, University Health Network, Toronto, ON, Canada; ^6^ Informatics and Biocomputing Program, Ontario Institute for Cancer Research, Toronto, ON, Canada; ^7^ Department of Pharmacology and Toxicology, University of Toronto, Toronto, ON, Canada; ^8^ Department of Radiation Oncology, Princess Margaret Cancer Centre, University Health Network, Toronto, ON, Canada; ^9^ Department of Radiation Oncology, University of Toronto, Toronto, ON, Canada; ^10^ Division of Medical Oncology, University of Toronto, Toronto, ON, Canada; ^11^ Department of Otolaryngology, University of Toronto, Toronto, ON, Canada

**Keywords:** microRNA, Nasopharyngeal Carcinoma, Distant Metastasis, Prognosis

## Abstract

**Purpose:**

Despite significant improvement in locoregional control in the contemporary era of nasopharyngeal carcinoma (NPC) treatment, patients still suffer from a significant risk of distant metastasis (DM). Identifying those patients at risk of DM would aid in personalized treatment in the future. MicroRNAs (miRNAs) play many important roles in human cancers; hence, we proceeded to address the primary hypothesis that there is a miRNA expression signature capable of predicting DM for NPC patients.

**Methods and results:**

The expression of 734 miRNAs was measured in 125 (Training) and 121 (Validation) clinically annotated NPC diagnostic biopsy samples. A 4-miRNA expression signature associated with risk of developing DM was identified by fitting a penalized Cox Proportion Hazard regression model to the Training data set (HR 8.25; *p* < 0.001), and subsequently validated in an independent Validation set (HR 3.2; *p* = 0.01). Pathway enrichment analysis indicated that the targets of miRNAs associated with DM appear to be converging on cell-cycle pathways.

**Conclusions:**

This 4-miRNA signature adds to the prognostic value of the current “gold standard” of TNM staging. In-depth interrogation of these 4-miRNAs will provide important biological insights that could facilitate the discovery and development of novel molecularly targeted therapies to improve outcome for future NPC patients.

## INTRODUCTION

Nasopharyngeal carcinoma (NPC) has a unique set of etiological, epidemiological and biological characteristics that renders it distinct from other epithelial malignancies of the head and neck region. The primary curative treatment for NPC is radiotherapy (RT) for patients with early stage disease, and concomitant chemoradiotherapy (CRT) for those with locally advanced disease. Technical improvements in RT delivery such as intensity-modulated radiation therapy (IMRT), and increasing use of CRT have led to significantly improved loco-regional control (LRC) for NPC patients, with 5-year LRC rates now well over 90% [1]. However, distant metastasis (DM) rates have not improved, and late metastatic disease remains the major cause of mortality in this patient population [1]. Currently, TNM staging is the primary tool utilized clinically to prognosticate outcomes for NPC patients. Other indicators such as tumor volume [2], plasma Epstein–Barr Viral (EBV) DNA titre [3, 4], and levels of expression of various proteins and transcripts [5–7] have been reported to correlate with clinical outcome; to date however, none has been universally adopted in the clinical management of NPC patients.

MicroRNAs (miRNA) are small non-protein-coding RNA molecules that function to reduce the expression of protein coding genes at the post-transcriptional level. Since their identification in 1993 [8], miRNAs have emerged as key regulators of gene expression in nearly all biological processes, including cancer [9]. Additionally, miRNA expression signatures associated with prognosis have been identified in numerous malignancies [10]. Hence, we undertook to identify and validate a miRNA expression signature capable of predicting for DM in NPC patients.

## RESULTS

### Generation and validation of miRNA signature associated with risk of distant metastasis

Using the Lasso method to fit a penalized Cox Proportional Hazards (PH) model to miRNA expression data from the Training cohort yielded 33 variables (miRNAs) with non-zero coefficients (Table S1). The 4 miRNAs most strongly associated with risk of DM (greatest absolute coefficient values) were combined with their coefficients within the penalized model to yield the following equation:

$$\begin{array}{lll} Risk\;Score & = & miR-154-5p_{\text{Expression}}*0.417+miR-\\ & & 449b-5p_{\text{Expression}}*0.280\\ & - & miR-140-5p_{\text{Expression}}*0.653–miR-\\ & & 34c-5p_{\text{Expression}}*0.311 \end{array}$$

**Figure 1:**
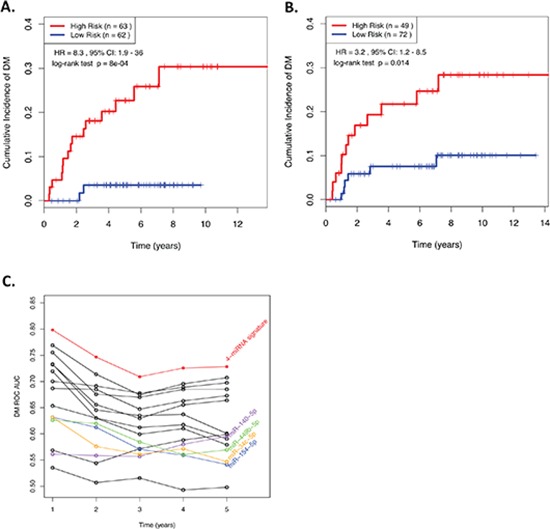
**(A&B) Kaplan-Meier curves showing NPC patients dichotomized based on risk score in** (A) the training cohort; and (B) the validation cohort. “High Risk” is defined as a RS ≥ the median in the training cohort, and “Low Risk” is defined as a RS < the median in the training cohort. **(C)** ROC AUCs across various time points demonstrating the ability of prognostic models generated using all possible combinations of 1, 2, 3, or 4 miRNAs from the 4-miRNA signature to predict distant relapse in NPC patients. RS, Risk Score; HR, Hazard Ratio; CI, Confidence Interval; ROC, receiver operating characteristic; AUC, Area Under the Curve.

The RS was calculated for each patient in the Training cohort, allowing the patients to be dichotomized into either a “low risk” (< median), or a “high risk” (≥ median) group. A highly significant relationship was observed between the RS and the likelihood of DM whether the RS was treated as a continuous (Wald test; HR = 2.76, Scaled HR; 5.65; *p* = 2.8 × 10^−5^), or a binary (log-rank test: HR = 8.25; *p* = 8.0 × 10^−4^) variable (Figure 1A).

When the same miRNA signature equation was applied to the independent Validation cohort of 121 NPC patients diagnosed approximately a decade earlier, a similar relationship was observed (Figure 1B). When treated as a continuous predictor, the scaled HR was 2.27 (raw HR: 1.7) with a Wald test *p*-valu*e* = 0.05. When dichotomized by the median from the training cohort, the HR was 3.2, with a log-rank test *p*-value of 0.014. These results indicated that there might be a greater benefit to utilizing this RS as a binary variable, with an established cut-point, as opposed to a continuous spectrum of risk. In addition, when all possible combinations of 1, 2, 3, or all 4 miRNAs from the original model were compared, the 4-miRNA signature consistently demonstrated the best performance across all time points (Figure 1C), underscoring the robust prognostic power of this 4-miRNA signature.

When the 4-miRNA signature was examined further in miRNA-Seq expression data generated by The Cancer Genome Atlas (TCGA), there was a significant difference observed in disease-specific survival for patients with a “high risk” score compared to those with a “low risk” score (HR = 1.8; *p* = 0.02; Figure S1A). For comparison, disease-specific survival in our dataset (combined training/validation, *n* = 246) is also illustrated in Figure S1B (HR = 1.9; *p* = 0.03; Figure S1A). Given that these data were generated from HNCs other than NPC (majority were oral cavity and larynx cancers), these results indicated that this 4-miRNA signature might be a useful prognostic tool across multiple tumours of the head and neck region.

### Multivariate analysis

**Figure 2:**
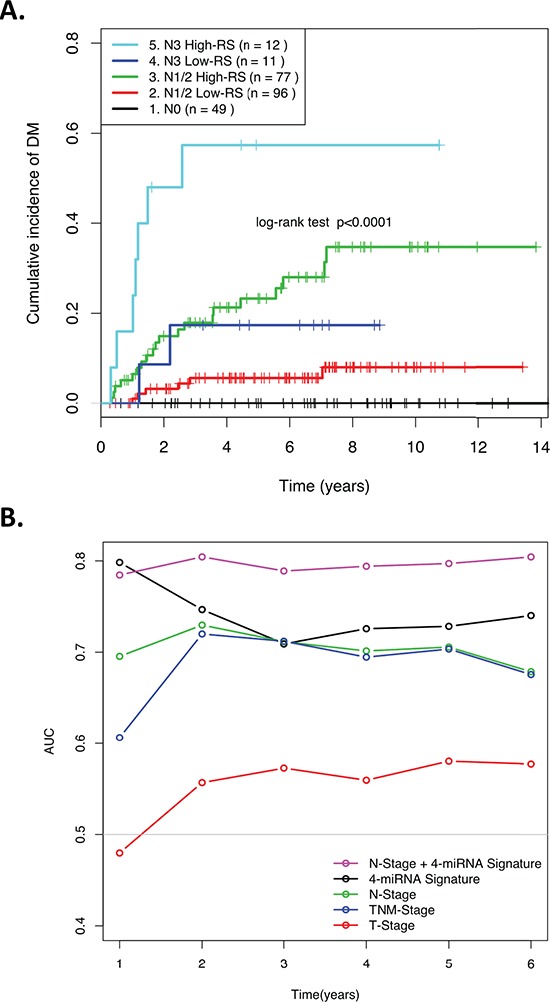
**(A)** Kaplan-Meier curve showing patients assigned to risk groups based on the combined N-stage and 4-miRNA signature Risk Score. **(B)** ROC AUCs over time demonstrating the ability of various clinical factors and the 4-miRNA signature RS to predict distant relapse in NPC patients.

Table 2 demonstrates that after controlling for clinical factors such as stage, age, gender, and treatment, the miRNA signature RS remained significantly associated with risk of DM (HR = 4.0; *p* = 7.3 × 10^−4^). Similar results were observed when only the training set was used for multivariate analysis (HR = 3.4; *p* = 0.02; Table S2). Nodal stage was the only other factor significantly associated with DM on both univariate and multivariate analyses (Table 2). For this reason, the 4-miRNA signature RS was then combined with N-stage to define five risk groups, based on the visual inspection of the distant metastasis-free survival (DMFS) Kaplan-Meier curves stratified by N-stage (Figure S2). Figure 2A demonstrates that when patients were stratified into five distinct groups, as a combination of nodal stage and RS, the patients in the N1/N2 and the N3 Groups were respectively further dichotomized by the miRNA RS, underscoring the ability of this 4-miRNA signature to provide improved risk prediction for DM in these clinically intermediate and high-risk groups. This improved prognostic ability was further corroborated when the area under the ROC curve was calculated as a function of follow-up time, and compared with clinical factors, the original 4-miRNA RS, and the 5-group N-Stage/miRNA risk-stratification (Figure 2B). The area under the ROC curves was consistently greatest in this 5-group stratification (except for Year-1, when the 4-miRNA signature alone marginally outperformed the 5-group model). C-statistics for each CoxPH model were also calculated for the combined data (*n* = 246), Training (*n* = 125) and Validation (*n* = 121) sets, further demonstrating the significantly greater prognostic value for this combined parameter (C-statistics; 0.78, 0.83, and 0.74, respectively), compared to all other models (Table S3).

**Table 1:** Clinical characteristics of the patients in the two independent cohorts

	Training Set (Dx ’00–’09; *n* = 125)	Validation Set (Dx ’93–’00; *n* = 121)	*p*-value^*^
Age (years)			
median	52	48	
range	14–89	16–79	0.30
Frequency (%)			
Gender			
Male	86 (67)	56 (71)	
Female	39 (33)	23 (29)	0.52
T stage			
T1	37 (30)	37 (31)	
T2	20 (16)	30 (25)	
T3	28 (22)	24 (20)	
T4	39 (31)	28 (23)	0.26
Unable to evaluate	1 (<1)	1 (<1)	
N stage			
N0	25 (20)	24 (20)	
N1	33 (26)	50 (41)	
N2	52 (42)	38 (31)	
N3	15 (12)	8 (7)	0.05
Unable to evaluate	none	1 (<1)	
TNM Stage			
I (%)	11(8)	10 (8)	
II (%)	22 (18)	37 (31)	
III (%)	41 (33)	39 (32)	
IV (%)	51 (41)	34 (28)	0.07
Unable to evaluate	none	1 (<1)	
Treatment			
Radiation only	34 (15)	86 (71)	
Radiation + chemo	91 (73)	35 (29)	1.43E-11
5-year Survival %			
Survival			
Overall	83%	73%	0.22
Disease-Free	72%	66%	0.35
Local Relapse-Free	90%	76%	0.002
Nodal Relapse-Free	94%	88%	0.08
Distant Relapse-Free	87%	87%	0.89

* Statistical tests: Wilcoxon rank-sum test (Age), Chi-squared test (Gender, T/N/TNM-stage, Treatment), log-rank test (all survival endpoints).

**Table 2:** Univariate and multivariate CoxPH analysis of clinical factors and miRNA-signature risk-score in the combined dataset from both the training and validation cohorts (*n* = 242)

	Univariate		Multivariate	
	HR (95% CI)	*p*-value	HR (95% CI)	*p*-value
**MiRNA RS (High Risk *vs*. Low Risk)**	4.5 (2.04–9.90)	1.9 × 10^−4^	4.0 (1.8–8.9)	7.3 × 10^−4^
**T stage (T1&2 *vs*. T3&4)**	1.7 (0.86–3.3)	0.13	1.5 (0.70–3.2)	0.30
**N stage (N0&1 *vs*. N2&3)**	2.5 (1.2–5.0)	8.0 × 10^−3^	2.7 (1.2–5.7)	0.01
**Age**	1.0 (0.98–1.0)	0.86	1.0 (0.98–1.0)	0.93
**Gender (Female *vs*. Male)**	1.31 (0.60–2.9)	0.50	1.3 (0.57–2.9)	0.53
**Chemotherapy (− *vs*. +)**	0.97 (0.5–1.9)	0.94	0.46 (0.21–1.0)	0.05

**Figure 3:**
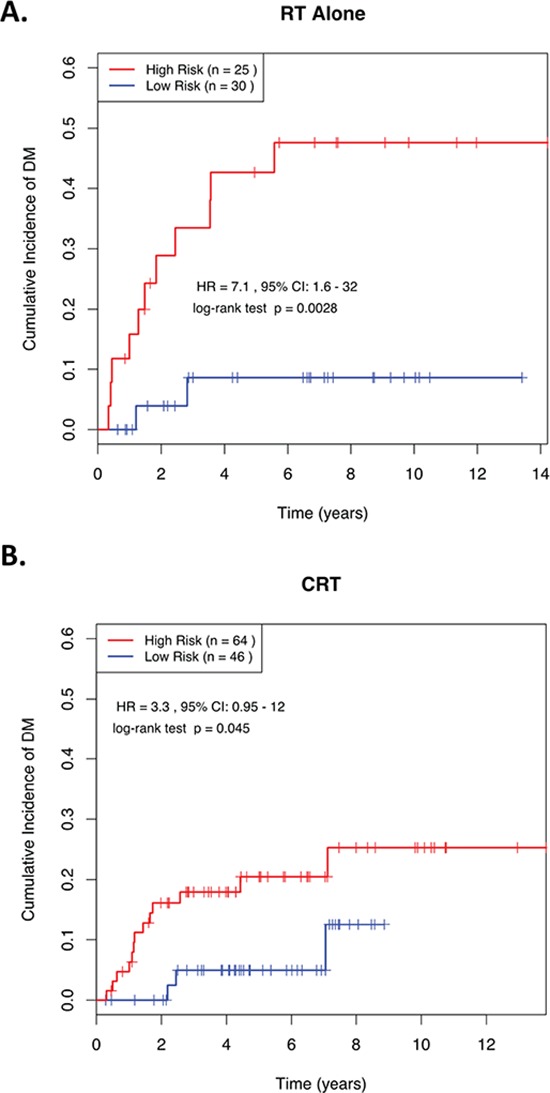
**Kaplan-Meier curves showing distant relapse in NPC patients dichotomized based on miRNA risk score in advanced stage patients (Stage III/IV) treated with** (A) RT alone or (B) combined CRT. ROC, receiver operating characteristic; AUC, Area Under the Curve; RT, radiotherapy; CRT, chemoradiotherapy; HR, Hazard Ratio; CI, Confidence Interval. RS, Risk Score; “High Risk” is defined as a RS ≥ the median in the training set, and “Low Risk” is defined as a RS < the median in the training set.

Figure 3 demonstrates the ability of this 4-miRNA signature to predict risk of DM in advanced stage (III & IV) patients who were treated with RT alone (HR = 7.13; *p* = 0.003; Figure 3A), as well as those who were treated with CRT (HR = 3.35; *p* = 0.045; Figure 3B). These data suggest that even in NPC patients with locally-advanced disease, this 4-miRNA signature could potentially identify a low-risk group of patients, whose likelihood of developing DM at 5-years was so low (<10%), that adding CT to their RT might have no benefit to their survival (Figure 3A). Conversely, there remained a high-risk group, whose outcome could be potentially improved with the administration of combined CRT, since RT alone was associated with a 5-year risk of 45% in developing DM (Figure 3A), *vs*. 20% when treated with CRT (Figure 3B).

### Random miRNA signatures

The distributions of hazard ratios and *p*-values (log-rank test) were plotted for 90,298 miRNA signature (90,000 randomly generated miRNA-signatures and 298 individual miRNAs) and compared with that of our 4-miRNA signature and an independent 5-miRNA signature generated by Liu *et al*. [11] (Figure S3). When compared with these random miRNA-sets, the log-rank *p*-value for the 4-miRNA signature described herein within the validation set (*p* = 0.014) was in the lowest 2.8% with its hazard ratio (HR = 3.2) in the highest 4.9% (absolute values). The signature described by Liu *et al.* performed slightly worse with its *p*-value in the lowest 9.7% and its HR in the top 10% (Figure S3).

In order to provide a more global perspective on the biological impact of this 4-miRNA signature, we identified miRNAs that were enriched in the random miRNA-sets that were significantly associated with distant metastasis; that is, signatures with *p*-values the same or lower than that of our 4-miRNA signature (*p* ≤ 0.014). The number of times each miRNA occurred in these signatures were then counted, normalized to the number of miRNAs in the signature, and then this distribution was plotted (Figure S4). The 15 miRNAs that appeared most frequently in signatures significantly associated with DM (top 5%) were then selected for further analysis (Figure S4).

### Validated miRNA target pathway enrichment analysis

**Figure 4:**
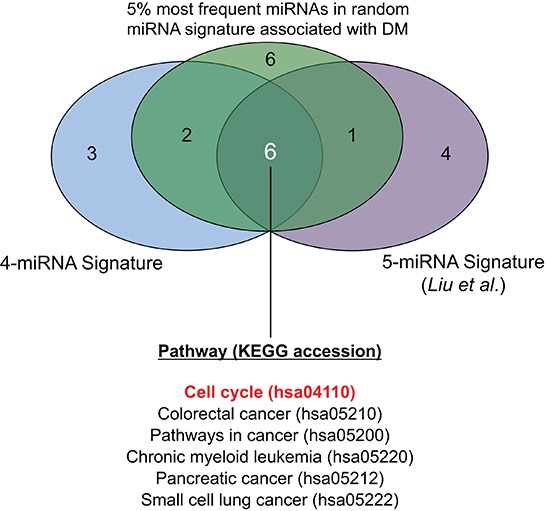
Venn diagram showing commonly and uniquely enriched pathways across three sets of miRNA-targets

**Figure 5:**
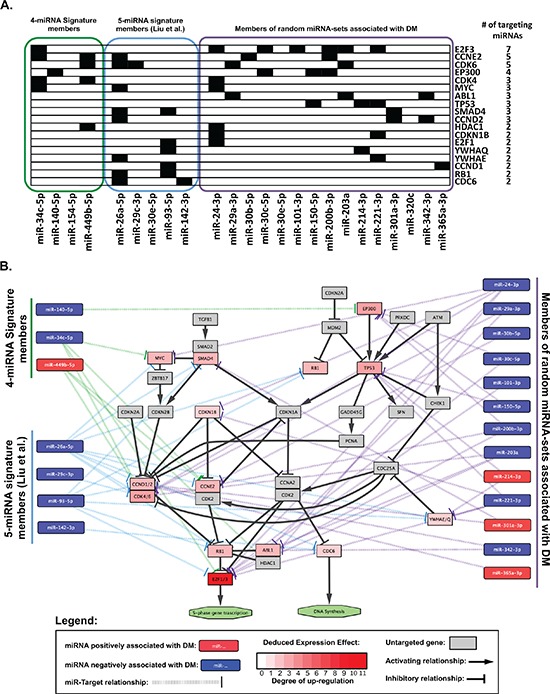
Cell-cycle related genes targeted by miRNAs associated with risk of distant metastasis **(A)** Chart showing Cell-cycle related genes targeted by at least 2 of the miRNAs queried. Black boxes indicate a miRNA-target relationship. **(B)** Pathway diagram modified from the “Cell-Cycle” KEGG pathway using Cytoscape (v3.1.1) showing validated targets of miRNAs from three prognostic groups. Note: miRNAs with no validated “cell-cylce” targets (miR-154–5p from the 4-miRNA signature and miR-30e-5p from The 5-miRNA signature) were omitted from the figure. See legend for further details.

The miRTarBase database was queried using a) the 4 miRNAs in the prognostic signature identified herein, b) the 5 miRNAs described by Liu *et al* [11], and c) the 5% most frequently occurring miRNAs in random signatures significantly associated with distant metastasis. This yielded a) 52 b) 888 and c) 1557 miR-target relationships (Table S4). When pathway enrichment analysis was performed, several biological pathways were observed to be significantly enriched (FDR < 0.05) within the targets of these three sets of miRNAs (Table S5). When significantly enriched pathways (FDR < 0.05) were compared amongst these three sets, six common pathways were identified (Figure 4). These included five generic “cancer” pathways (colorectal, chronic myeloid leukemia, pancreatic, small cell lung cancers, and one “pathways in cancer”), and one functional pathway: the “Cell Cycle” KEGG pathway (accession: hsa04110). Given that only one miRNA overlapped among the three sets analyzed (miR-30e), these data provided strong indications that cell cycling and proliferation were important biological processes mediating the distant metastasis underlying these miRNAs. Therefore, we proceeded to investigate the cell cycle-related targets which were common to multiple miRNAs, and observed that 18 members of the cell cycle pathway were in fact targeted by multiple (at least 2) miRNAs contained in the three sets of signatures analyzed (Figure 5A). Figure 5B illustrates these 18 genes in the context of the cell cycle pathway, with a *Deduced Expression Effect* (DEE) denoted by the colour of each miRNA-target node. These values were calculated using the following formula:

$$\begin{array}{l} \text{DEE}=\text{the number of targeting miRNAs }negatively\text{ associated with DM}\\ \;\;\;-\text{the number of targeting miRNAs }positively\text{ associated with DM} \end{array}$$

Thus, given the inhibitory relationship between miRNAs and their targets, we would anticipate that those genes with a higher DEE would be up-regulated in patients at high risk of DM, whereas those with a lower DEE would be down-regulated in high risk patients for DM. When DEEs were calculated, all 18 targets had positive values (indicating an expected increase in expression), likely reflecting an enrichment in miRNAs which were negatively associated with DM (Figure 5B). These results suggest that the cell cycle pathway is activated in patients at risk of DM. In particular, key regulators of the cell cycle such as the cyclins (CCND1/D2), cyclin-dependent kinases (CDK4/6) and S-phase promoting transcription factors (E2F1/3) appeared to be particularly important, as indicated by their respective DEEs.

## DISCUSSION

Given the continual challenge of DM in NPC patients, the validated prognostic 4-miRNA signature presented herein could prove to be a valuable tool in guiding treatment decisions for these patients. To date, there are no clinically utilized prognostic biomarkers in NPC; plasma levels of EBV DNA titres have certainly been associated with clinical outcome, particularly in predicting risk of relapse when the titre remained elevated, or increased post-treatment [3, 4]. However, this has not contributed to treatment decisions, which to date, have relied on TNM staging, with administration of combined CRT for patients with locally-advanced disease. However, such CRT regimens are also associated with significant acute and long-term morbidities and even mortality, diluting any potential gains with these aggressive treatments [12–14]. Hence, the utility of this 4-miRNA signature, which has been validated in an independent cohort (Figure 1) and demonstrated to add to the prognostic value of the TNM staging category (Figure 2), is extremely promising. Furthermore, the apparent ability of this 4-miRNA signature to identify a low-risk group of patients with locally-advanced NPC, who could be cured with RT alone (Figure 3), suggests a potential predictive value of this signature, which again to date, has never been previously documented. However, given the inherent risk of bias in retrospective studies, in order to confirm the ability of this signature to predict treatment success for patients treated with RT alone, further verification in an additional independent cohort of randomized controlled trial (RCT) participants randomly designated to RT alone *vs*. CRT would be required. The successful demonstration of such a predictive miRNA set would potentially facilitate the de-escalation for patients with truly a favourable prognosis, thereby sparing them the increased toxicity of combined CRT. In contrast, patients at high-risk will still have a 60% probability of developing DM at 5-years, despite CRT; for this group of patients, the need to identify and evaluate novel therapeutics cannot be over-emphasized. Indeed, identifying such targetable pathways could be achieved by interrogating the underlying biology of these miRNA signatures.

There exists another validated prognostic 5-miRNA signature set for NPC, generated by a group from Guangdong, which was associated with overall, disease-free, and DMFS [11]. We have evaluated their 5-miRNA signature, compared to either our 4-miRNA signature alone, or each combined with N-stage, and our signatures appeared to demonstrate a more robust performance, based on direct comparisons and a ROC curve analysis (Figure S5, and Table S4). Specifically, comparing the HR of our 4-miRNA signature to the HR of the 5-miRNA signature for DM (Figure S5A *vs*. S5B), our current 4-miRNA signature appeared to perform better, with a higher HR and greater statistical significance. Similarly, the greatest AUC in the ROC analysis was observed with our 4-miRNA signature combined with N-stage (Figure S5C). However, given significant technical, clinical and geographical differences between our two studies, it is impossible to draw any conclusions regarding the superiority of one or the other signature at present. Also of note, is the fact that there is absolutely no overlap between their 5-miRNAs with our 4-miRNAs. This is not surprising, again given the differences in platforms (in-house *vs*. Nanostring nCounter), different population cohorts, as well as redundancy in biological processes. This phenomenon has been similarly observed with other miRNA signatures [15, 16], as well as mRNA signatures [17]. Future analysis on additional independent datasets would be required to definitively determine the optimal signature, as well as the potential importance of geographical and population differences.

Preliminary pathway enrichment analysis indicated a role in cell cycle regulation not only for the 4 miRNAs in this signature set, but also for those in the signature identified by Liu *et al.* [11] as well as miRNAs that were over-represented in random miRNA-sets observed to be significantly associated with DM (Figure 4). Using a simple formula to deduce the potential effect of miRNAs positively or negatively associated with DM on their putative targets, we observed several important mediators of the cell cycle to be up-regulated in patients at high risk of DM, attributed to down-regulation of miRNAs which target these genes (Figure 5B). In particular, the cyclins (CCN) D1 and D2, cyclin-dependent kinases (CDK) 4 and 6, and E2F transcription factors 1 and 3 were targeted by a number miRNAs which were negatively associated with distant metastasis in our data sets. CCND1 has already been well-established to play an important role in NPC development and progression [18–20]; however a link between CCND1 with clinical outcome has yet to be reported. Interestingly, using immunohistochemistry, two groups have recently demonstrated that high CDK4 expression was associated with poor clinical outcome in NPC patients; including increased local and distant recurrence [21, 22]. Moreover, cyclins D & E, E2F1 and CDK4 and 6, have all been previously observed to be associated with poor outcome in a number of other tumour types [23–27]. These data suggest a potentially important role for markers of cell cycle activation as potential prognostic indicators in NPC. This relationship between cell cycle activation and poor outcome/DM also indicates that cell cycle inhibition might be a useful therapeutic strategy in NPC.

Despite the numerous publications purporting the identification of prognostic miRNA signatures (reviewed by Nair *et al* [28]); none to date has been utilized in the clinic. We are hopeful that this 4-miRNA signature set will be verified and implemented, based on several strengths of this current NPC study: a) large sample size (both Training and Validation cohorts were in excess of 100 patients); b) uniformity of treatment at a single institution; c) identical DMFS in both cohorts despite the difference in time period and increased use of chemotherapy in the Training cohort; d) a single experimentalist to ensure technical consistency; e) the same type of tissues (both cohorts were FFPE blocks); and f) consistency of the miRNA profiling platform. Indeed, if this 4-miRNA signature can be verified in yet a third independent RCT cohort, this signature could be theoretically readily translatable, given that FFPE processing is broadly utilized around the world.

In conclusion, we have successfully validated a 4-miRNA signature that can prognosticate for DM in patients with NPC. This signature adds to the prognostic value of the current “gold standard” of the TNM staging categories, and there is a suggestion of its potential predictive role in selecting NPC patients for de-escalating treatment to RT alone, despite locally-advanced disease at presentation. Further interrogation of the underlying biological pathways such as cell cycle and proliferation could render the selection of patients who might be sensitive to compounds such as CDK, HDAC or EGFR inhibitors. Such novel molecular targeted agents have been demonstrated to have promising efficacy in the clinic [29–31], as well as in pre-clinical NPC models [32]; the utilization of a prognostic miRNA signature would enrich for potentially sensitive patients, thereby improving clinical outcome for future patients with NPC.

## MATERIALS & METHODS

### Patients and samples

Approval for this study was obtained from the Institutional Research Ethics Board (REB) at the Princess Margaret Cancer Center (PM). Diagnostic formalin-fixed paraffin-embedded (FFPE) blocks were collected for NPC patients treated at the PM who were diagnosed with NPC between 2000–2009 (Training cohort), and 1993–2000 (Validation cohort) (Table 1). Patients with metastatic disease at the time of diagnosis were excluded. Diagnostic and follow-up data were collected through chart reviews; the clinical characteristics for these two patient cohorts are shown in Table 1. It is important to note that despite the greater utilization of CRT (which reflected clinical practice), and the superior LRC rates in the Training cohort, the 5-year distant metastasis-free survival (DMFS) rates for both cohorts was identical, at 87%.

Normal nasopharyngeal epithelial tissues (*n* = 17) were macro-dissected from FFPE blocks of patients who underwent a quadroscopy and were not diagnosed with NPC. These tissues were examined by at least one head and neck cancer (HNC) pathologist (BP-O or IW), and deemed to be free of malignant cells.

### MiRNA expression profiling

A representative section from each block was stained with hematoxylin and eosin (H&E) and reviewed by a HNC pathologist (BP-O) to identify regions containing malignant cells. Samples were then macro-dissected to ensure that > 70% of the material analyzed was malignant epithelium. Specimens were de-paraffinized with xylene, and total RNA was extracted using the Recover All Total Nucleic Acid Isolation Kit for FFPE (Ambion, Inc), according to the manufacturer's instructions. RNA was quantified and purity-assessed using a Nanodrop spectrophotometer (Thermo Scientific). No significant relationship was observed between the time of sample fixation (at diagnosis) and either quality or quantity of RNA extracted (data not shown). Two hundred nanograms of total RNA from each sample was analyzed according to the manufacturer's instructions using the nCounter^®^ Human miRNA Assay from Nanostring (v1.0) to measure 734 unique human and viral miRNAs. Data were normalized using the NanoStringNorm [33] package (v1.1.13) in the ‘R’ statistical computing environment (v2.15.2). Raw counts were background-corrected by subtracting the mean + 2 standard deviations of the negative control probes included in the assay, followed by variance stabilization and normalization (vsn [34]) called through the NanoStringNorm package.

### Statistical analysis

All statistical tests were performed in the ‘R’ statistical computing environment (v2.15.2), with the exception of pathway enrichment analysis. MiRNA expression in tumour *vs*. normal samples was compared using Welch's *t*-tests with the Benjamini & Hochberg false discovery rate (FDR) correction for multiple comparisons [35]. Prior to all regression analysis, the data were standardized (mean centered and standard deviation scaled), using the R package ‘ffmanova’ [36], to maintain consistency across datasets. In order to identify a signature associated with risk of DM, an L1 penalized (Lasso [37]) Cox proportional hazard (CoxPH) regression model was fitted to the Training cohort data using the penalized (v0.9–42) package [38] in R. The Lasso algorithm performed poorly when the proportion of 0s (expression below background) was high, so a cut-off of > 80% non-zero values across samples in the training set was used to filter miRNAs to include in the model generation; resulting in 298 miRNAs remaining. Leave-one-out cross validation was used to determine the L1 tuning parameter that yielded the highest log-likelihood, and a penalized CoxPH regression model was fitted using this optimal value. After fitting, the 4 miRNAs most strongly associated with DM were selected for inclusion in the risk score (RS) equation in order to maintain the number of events per variable (EVP) at ~five [39]. The final equation was generated using the regression coefficients from the CoxPH model. RS was calculated for each patient in the Training cohort, and patients were dichotomized to “high risk” (RS ≥ median) or “low risk” (RS < median) groups. The difference in DMFS between the two groups was compared using the log-rank test whereas the RS was analyzed as a continuous predictor using the CoxPH model and the Wald-test. For the continuous HRs, a scaled HR (D Index) [40] was also calculated using the D.index function in survcomp package (v1.12) to provide a closer comparison with HRs from binary analyses. RS and risk-groups were determined similarly for the Validation cohort, with the cut-point maintained as the median RS in the Training cohort and DMFS compared using the log-rank and Wald tests as previously mentioned.

In order to determine whether all 4 miRNAs are integral to the prognostic ability of the signature, all possible combinations using 4, 3, 2, or 1 of the miRNAs from the model (15 sets in total) were compared based on the AUC of their respective DM ROC curves, across multiple time points; calculated using the ‘survivalROC’ package (v1.0.3) [41].

Multivariate analysis was performed by including the 4-miRNA signature risk-group assignment (“high risk” or “low risk”) for each patient adjusted with clinical variables (T-Stage, N-Stage, gender, age, treatment) in a CoxPH model fitted to the combined Training and Validation datasets (*n* = 242; one patient removed due to unevaluable nodal stage, three patients removed due to unevaluable tumour stage). The two variables observed to be significantly associated with DM in this model (the 4-miRNA signature RS, and N-stage) were then combined to create five risk groups: 1. N0; 2. N1/2 & Low-RS; 3. N1/2 & High-RS; 4. N3 & Low-RS; and 5. N3 & High-RS. The area-under the curve (AUC) was calculated for the receiver operating characteristic (ROC) curves across multiple time points using the ‘survivalROC’ package (v1.0.3) [41] and the C-statistics for various predictive models were calculated using the ‘survConcordance’ function in the ‘survival’ package (v2.37–4) [42] for R. A depiction of the signature generation and validation workflow is depicted in Figure S6.

### Random miRNA signatures

It has been demonstrated that particular disease types and data sets are prone to random prognostic relationships beyond what would be expected by random chance [43–45]. In order to address this potential issue in our own data set, 10,000 random miRNA sets for each size from 2–10 members (total = 90,000) were selected from the pool of miRNAs expressed above background in > 80% of samples (298 miRNAs). A CoxPH model was then fitted to the Training cohort data using each set of randomly selected miRNAs with DM as the endpoint. A Risk Score was calculated using the coefficients from the model, and high *vs*. low risk patients (dichotomized using the median RS in the Training set) were then compared in the Validation set using the log-rank test. The resulting *p*-values and HRs from these randomly-generated signatures as well as all individual miRNAs included in the analysis (totaling 90,298 signatures) were then compared to our 4-miRNA signature, as well as the 5-miRNA signature generated by Liu *et al* [11]. Signatures with *p*-values the same or lower than that of our 4-miRNA signature (*p* = 0.014) were then interrogated to determine which miRNAs appeared most frequently within signatures significantly associated with a risk of DM. The number of occurrences was tabulated and normalized to the number of miRNAs in the signature; the top 5% most frequently occurring miRNAs were then selected for further pathway enrichment analysis.

### TCGA data retrieval and analysis

Level 3 (Reads-per-million; RPM) miRNA-Seq and Level 1 clinical data were downloaded from the Broad Institute Firehose (stddata run 2013_11_14) repository for data generated by TCGA. At the time of analysis, there were 260 HNSCC samples with miRNA-seq values and sufficient follow-up data to determine disease specific survival status. RPM data were log_2_ transformed and standardized (mean centered, standard deviation scaled) before Risk Scores were calculated using the 4-miRNA equation described above. Patients were dichotomized into “low risk” (<median RS) *vs*. “high risk” (≥median RS) groups, and then compared using the log-rank test.

### Target identification and pathway analysis

Validated targets of the 4 miRNAs in our signature, the 5 miRNAs in the signature generated by Liu *et al* [11], and the top 5% most frequently occurring miRNAs in random signatures significantly associated with distant metastasis (Figure S4) were downloaded from the manually curated database of validated miRNA targets, miRTarBase (http://mirtarbase.mbc.nctu.edu.tw; release 4.5) [46]. The union of the targets for each miRNA set was inputted into the Database for Annotation, Visualization and Integrated Discovery (DAVID; v6.7) [47] for pathway enrichment analysis. For all DAVID analyses, KEGG, Panther, Reactome and Biocarta databases were queried. To ensure that the enrichment observed was not due to bias in the validated target database, a complete list of validated targets for all human miRNAs was downloaded from the miRTarBase database, and used as the background for enrichment analysis.

## SUPPLEMENTARY TABLES AND FIGURES




