# Supplementary material for: Identification of a microRNA signature associated with risk of distant metastasis in nasopharyngeal carcinoma
Source: Oncotarget. 2015 Jan 23;6(6):4537–50. doi: 10.18632/oncotarget.3005 (PMC4414210; doi:10.18632/oncotarget.3005)
Supplement: Supplementary file 1 [file oncotarget-06-4537-s001.pdf]

SUPPLEMENTARY TABLES AND FIGURES

Supplementary Table S1: MiRNAs with non-zero coefficients in the penalized regression model fitted to miRNA expression data from the training set

miRNA	Penalized Coefficient
hsa-miR-140–5p	−0.65265805
hsa-miR-154	0.416841936
hsa-miR-34c-5p	−0.311288747
hsa-miR-449b	0.27970981
hsa-miR-136	0.278252522
hsa-miR-1246	0.252954521
hsa-miR-31	0.246736327
hsa-miR-633	−0.232982777
hsa-miR-375	0.227348203
hsa-miR-646	−0.187297587
hsa-miR-188–5p	−0.184079758
hsa-miR-574–3p	0.167259093
hsa-miR-542–3p	−0.165407907
hsa-miR-1	0.153553988
hsa-miR-561	−0.152880207
hsa-miR-580	−0.147656138
hsa-miR-155	−0.145379233
hsa-miR-34c-3p	0.114592858
hsa-miR-196a	−0.099335183
hsa-miR-296–5p	0.094110203
hsa-miR-107	0.093446126
hsa-miR-663b	0.074681673
hsa-miR-600	0.063613683
hsa-miR-142–5p	−0.060944673
hsa-miR-132	−0.056635448
ebv-miR-BART14	0.048490936
hsa-miR-362–5p	0.043422478
hsa-miR-377	0.04287268
hsa-miR-934	−0.036495034
hsa-miR-144	0.034527264
hsa-miR-188–3p	−0.033462301
hsa-miR-93	0.007480249
hsa-miR-579	−0.003676771

A.

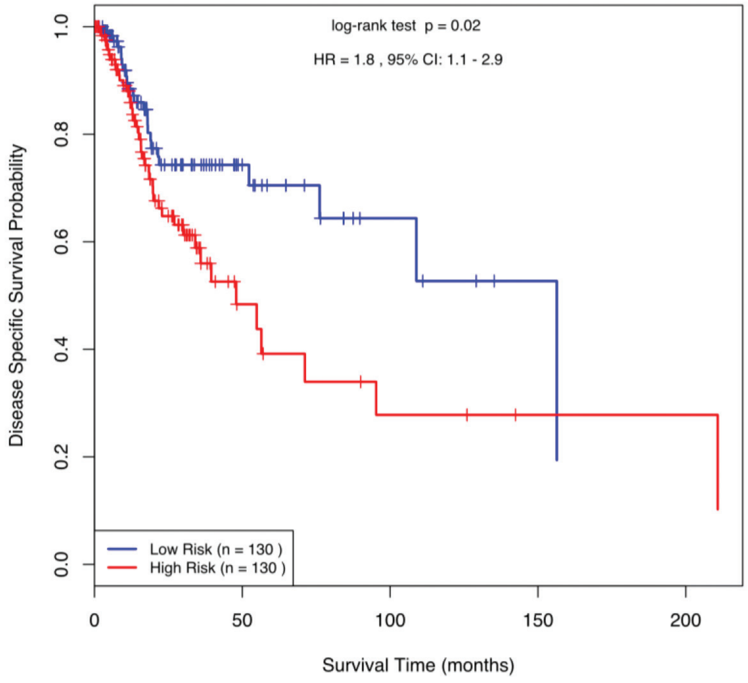

B.

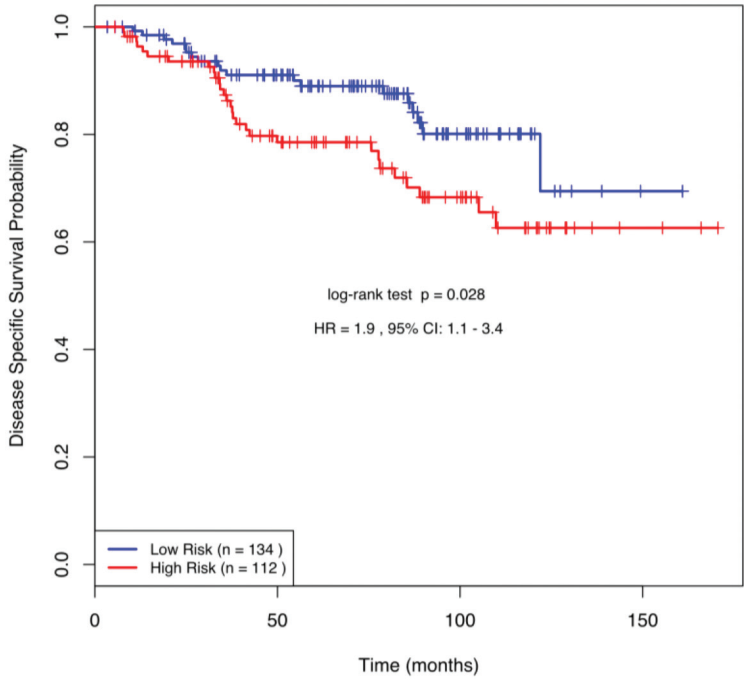

Figure S1: Kaplan-Meier curves demonstrating the association between Disease Specific Survival the 4-miRNA signature applied to **A)** HNSCC miRNA-Seq Data generated by TCGA (n=260) and **B)** the combined training and validation sets of NPC patients (n=246). “High Risk” is defined as a RS ≥ the median, and “Low Risk” is defined as a RS < the median. RS; Risk Score, HR; Hazard Ratio, CI; Confidence Interval.

Supplementary Table S2: C-statistics for comparison of various CoxPH models' ability to predict DM

Covariates included in the model (levels for each variable)	C-Statistic ± SE		
	Combined Data	Training Set	Validation Set
4-miRNA Signature (High Risk/ Low Risk), N-stage (N0&1/ N2&3)	0.78 ± 0.048	0.83 ± 0.069	0.74 ± 0.065
5-miRNA Liu et al. Signature (High Risk/Low Risk), N-stage (N0&1/N2&3)	0.74 ± 0.048	0.79 ± 0.069	0.69 ± 0.066
4-miRNA Signature (High Risk/ Low Risk)	0.71 ± 0.050	0.77 ± 0.073	0.66 ± 0.070
TNM-stage (I/II/III/IV)	0.67 ± 0.049	0.80 ± 0.070	0.48 ± 0.067
N-stage (N0&1/N2&3)	0.62 ± 0.043	0.66 ± 0.063	0.59 ± 0.058
5-miRNA Liu et al. Signature (High Risk/Low Risk)	0.60 ± 0.044	0.59 ± 0.063	0.60 ± 0.061
T-Stage (T1&2/T3&4)	0.55 ± 0.043	0.55 ± 0.063	0.55 ± 0.059

Supplementary Table S3: All miRNA-Target entries from miRTarBase for miRNAs from our 4-miRNA signature, Liu et al.'s 5-miRNA signature and the 5% most frequently occurring miRNAs in random signatures significantly associated with distant metastasis

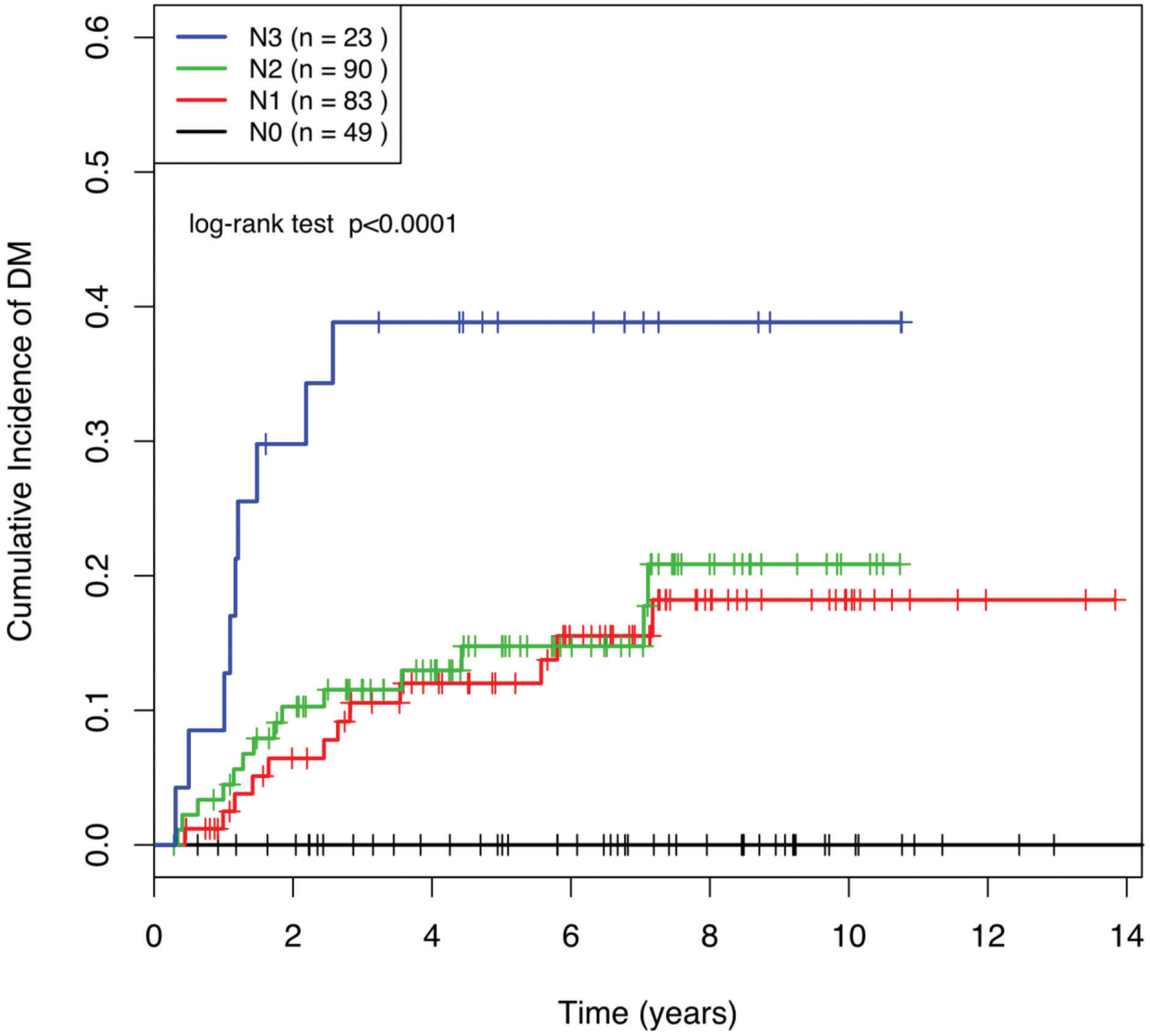

Figure S2: Kaplan-Meier curves demonstrating distant metastasis free survival (DMFS) based on clinical Nodal-stage in the combined dataset (n=245).

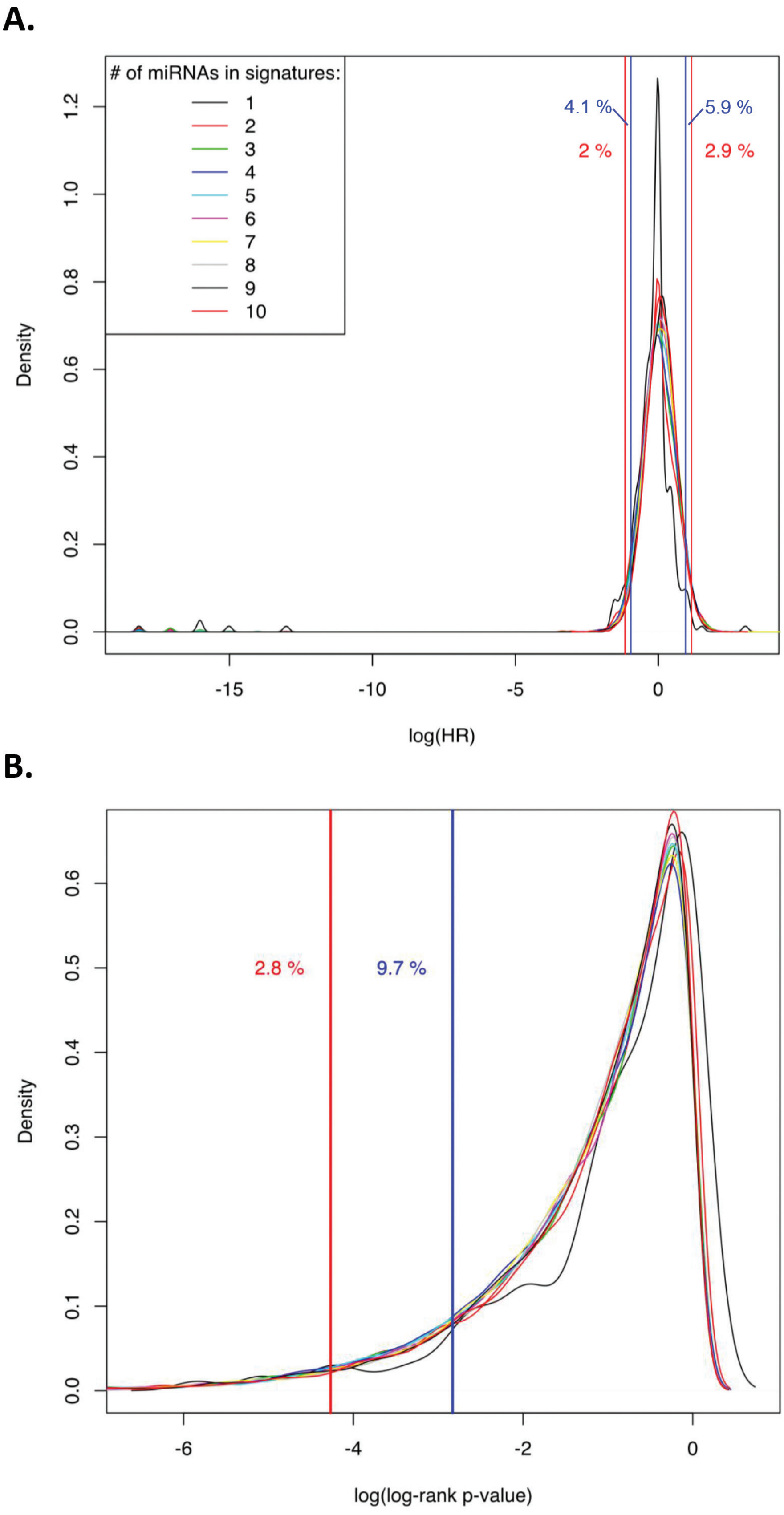

Figure S3: Distribution of **A)** hazard ratios and **B)** p-values for random miRNAs signatures. Red lines and percentages show the position of the 4-miRNA signature described herein and blue lines and percentages show the position of the 5-miRNA signature described by Liu *et al*.

Supplementary Table S4: Pathway enrichment results from DAVID for targets of the three sets of miRNAs analyzed

Category	Term	Count	Fold Enrichment	*P*-Value	FDR	Genes (Entrez IDs)
**4-miRNA Signature**						
KEGG_PATHWAY	hsa05200:Pathways in cancer	14	5.934944238	5.56E-08	5.51E-07	596, 5156, 2033, 2254, 1019, 1871, 1021, 9134, 7422, 2247, 3065, 7046, 4233, 4609
KEGG_PATHWAY	hsa05218:Melanoma	7	14.00438596	5.49E-06	5.44E-05	1021, 5156, 2247, 2254, 1019, 4233, 1871
KEGG_PATHWAY	hsa04110:Cell cycle	8	8.526034713	1.99E-05	1.97E-04	1021, 9134, 993, 2033, 3065, 1019, 1871, 4609
KEGG_PATHWAY	hsa05220:Chronic myeloid leukemia	6	9.636820926	2.60E-04	2.58E-03	1021, 3065, 1019, 7046, 1871, 4609
KEGG_PATHWAY	hsa05222:Small cell lung cancer	6	9.636820926	2.60E-04	2.58E-03	1021, 9134, 596, 1019, 1871, 4609
PANTHER_PATHWAY	P00059:p53 pathway	6	6.371312898	0.001374557	1.08E-02	9134, 993, 2033, 3065, 1871, 23411
KEGG_PATHWAY	hsa05212:Pancreatic cancer	5	8.771978022	0.001931006	1.90E-02	1021, 7422, 1019, 7046, 1871
KEGG_PATHWAY	hsa04330:Notch signaling pathway	4	13.03265306	0.00294798	2.88E-02	4855, 2033, 4851, 3065
KEGG_PATHWAY	hsa05210:Colorectal cancer	5	7.602380952	0.003271684	3.20E-02	596, 5156, 7046, 4233, 4609
KEGG_PATHWAY	hsa05219:Bladder cancer	4	11.6959707	0.00402467	3.92E-02	7422, 1019, 1871, 4609
KEGG_PATHWAY	hsa05215:Prostate cancer	5	7.127232143	0.004135801	4.02E-02	9134, 596, 5156, 2033, 1871
**5-miRNA Signature (Liu et al.)**						
**Category**	**Term**	**Count**	**Fold Enrichment**	***P*-Value**	**FDR**	**Genes (Entrez IDs)**
KEGG_PATHWAY	hsa05222:Small cell lung cancer	18	2.95435386	6.30E-05	0.000751692	595, 5728, 1869, 596, 1282, 1284, 4843, 5970, 5925, 6502, 3685, 1021, 9134, 10000, 3915, 898, 5296, 4609
KEGG_PATHWAY	hsa05200:Pathways in cancer	40	1.732830434	4.87E-04	0.005798685	3725, 1869, 6774, 5970, 2932, 332, 1026, 7422, 3320, 2956, 7048, 7046, 595, 5601, 1487, 5728, 1857, 596, 1282, 1284, 4843, 5925, 2737, 6502, 3685, 1021, 9134, 10000, 2885, 3915, 54361, 4089, 1613, 112399, 898, 5879, 3569, 998, 5296, 4609

(*Continued*)

KEGG_PATHWAY	hsa04110:Cell cycle	21	2.287093253	5.52E-04	0.00656649	595, 10971, 8881, 1869, 4174, 8697, 2932, 5925, 1026, 6502, 9232, 1021, 9134, 990, 894, 51433, 4089, 7531, 898, 4085, 4609
KEGG_PATHWAY	hsa05212:Pancreatic cancer	15	2.68921954	8.97E-04	0.01065014	595, 5601, 1869, 6774, 5970, 5925, 1021, 10000, 7422, 4089, 7048, 7046, 5879, 5296, 998
KEGG_PATHWAY	hsa05210:Colorectal cancer	16	2.486034063	0.001334521	0.015802398	3725, 595, 5601, 1857, 596, 2932, 332, 10000, 2885, 2956, 4089, 7048, 7046, 5879, 5296, 4609
REACTOME_PATHWAY	REACT_152:Cell Cycle, Mitotic	39	1.608088235	0.001937666	0.019112212	5717, 5718, 8881, 1869, 8697, 332, 6241, 5710, 1026, 5684, 5683, 81565, 3320, 6118, 57551, 595, 5116, 4174, 3619, 7283, 5426, 5983, 5693, 5925, 57122, 6502, 6233, 9232, 5687, 1021, 9134, 1639, 990, 5501, 894, 51433, 7531, 898, 4085
KEGG_PATHWAY	hsa05220:Chronic myeloid leukemia	15	2.46196155	0.002214934	0.026101979	595, 1487, 1869, 5970, 5925, 1026, 1021, 10000, 2885, 25759, 4089, 7048, 7046, 5296, 4609
BIOCARTA	h_g1Pathway:Cell Cycle: G1/S Check Point	9	3.459272727	0.002399096	0.029798975	595, 1021, 1869, 2932, 4089, 5925, 1026, 6502, 898
KEGG_PATHWAY	hsa04510:Focal adhesion	25	1.879562044	0.002594272	0.030509182	3725, 2932, 7422, 3696, 81, 595, 5601, 1281, 5728, 1282, 596, 1284, 858, 3685, 10000, 25759, 2885, 5501, 3915, 1277, 894, 1278, 5879, 5296, 998
REACTOME_PATHWAY	REACT_1538:Cell Cycle Checkpoints	19	2.057486631	0.003414782	0.033460376	5717, 8881, 5718, 4174, 8697, 5710, 5983, 5693, 1026, 6233, 5684, 5683, 5687, 9134, 990, 51433, 6118, 898, 4085

(Continued)

PANTHER_ PATHWAY	P00048:PI3 kinase pathway	16	2.185714286	0.00437582	0.046489727	595, 10971, 5728, 22887, 2309, 4843, 2932, 2776, 2771, 3481, 10000, 3484, 2885, 894, 7531, 5296

5% most frequently occurring miRNAs in random signatures significantly associated with distant metastasis

Category	Term	Count	Fold Enrichment	*P*-Value	FDR	Genes (Entrez IDs)
KEGG_ PATHWAY	hsa05210:Colorectal cancer	23	2.846472868	6.05E-06	7.29577E-05	3725, 595, 7855, 596, 83439, 5604, 91, 581, 4436, 332, 7157, 54205, 7040, 8322, 8323, 8312, 4089, 2353, 7046, 5879, 4609, 5296, 4087
BIOCARTA	h_g1Pathway:Cell Cycle: G1/S Check Point	14	3.6995	1.29E-05	0.00016645	595, 1869, 1029, 1019, 1027, 25, 7157, 983, 7040, 1719, 1021, 472, 3065, 4089
KEGG_ PATHWAY	hsa05200:Pathways in cancer	52	1.794285467	1.83E-05	0.000220756	3725, 1869, 332, 2335, 331, 3728, 8322, 8323, 387, 5743, 5604, 581, 2322, 25, 8453, 8312, 2113, 4089, 4792, 5879, 4609, 4087, 8030, 2033, 5970, 8031, 1029, 1027, 7040, 7422, 3320, 7046, 595, 7855, 596, 83439, 5898, 91, 4436, 7428, 1019, 1871, 7157, 1870, 1021, 9134, 54205, 3065, 2353, 3569, 5296, 999
KEGG_ PATHWAY	hsa05212:Pancreatic cancer	20	2.855992844	2.80E-05	0.000337833	595, 1869, 5898, 9459, 91, 5970, 5604, 1029, 1019, 1871, 7157, 1870, 1021, 7040, 7422, 4089, 7046, 5879, 5296, 4087
KEGG_ PATHWAY	hsa05220:Chronic myeloid leukemia	21	2.745373403	3.14E-05	0.000378112	595, 1869, 6464, 91, 5970, 5604, 1029, 1019, 25, 1027, 1871, 7157, 1870, 1021, 7040, 3065, 4089, 7046, 4792, 5296, 4609
KEGG_ PATHWAY	hsa04115:p53 signaling pathway	19	2.89110751	3.97E-05	0.000478735	595, 8493, 581, 1111, 1029, 27244, 6241, 1019, 7157, 5054, 983, 1021, 64326, 54205, 9134, 472, 894, 891, 900

(*Continued*)

KEGG_PATHWAY	hsa04110:Cell cycle	26	2.255433601	1.05E-04	0.00126249	1869, 2033, 8317, 1029, 1027, 983, 7040, 5111, 595, 4173, 890, 1111, 1019, 25, 1871, 7157, 1870, 9134, 1021, 472, 3065, 4089, 894, 891, 4609, 4087
KEGG_PATHWAY	hsa05222:Small cell lung cancer	18	2.353177203	0.001008706	0.012099305	595, 1869, 596, 5743, 5970, 2335, 1019, 331, 1027, 1871, 7157, 1870, 1021, 9134, 54205, 4792, 5296, 4609
KEGG_PATHWAY	hsa04310:Wnt signaling pathway	25	1.966520497	0.00127272	0.01524388	3725, 23236, 2033, 1454, 5534, 81839, 8322, 8323, 387, 85407, 595, 7855, 83439, 79718, 5566, 7157, 57680, 5529, 8312, 10725, 4089, 894, 5879, 4609, 4087
KEGG_PATHWAY	hsa05215:Prostate cancer	19	2.204469477	0.001565338	0.018718412	595, 1869, 596, 83439, 2033, 5604, 5970, 5170, 1385, 1027, 1871, 7157, 1870, 3481, 9134, 3320, 468, 4792, 5296
KEGG_PATHWAY	hsa05219:Bladder cancer	12	2.855992844	0.001953074	0.023305052	595, 1869, 7422, 9252, 5604, 1029, 1019, 1871, 7157, 4609, 1870, 999
PANTHER_PATHWAY	P04398:p53 pathway feedback loops 2	14	2.428571429	0.003000301	0.033324017	8493, 890, 1432, 1029, 8626, 5170, 7157, 1871, 983, 9134, 472, 900, 5296, 4609
BIOCARTA	h_ctcfPathway:CTCF: First Multivalent Nuclear Factor	8	3.523333333	0.003408536	0.043224237	7040, 10664, 1029, 7049, 4089, 1027, 4086, 7157
KEGG_PATHWAY	hsa05014:Amyotrophic lateral sclerosis (ALS)	11	2.836159561	0.003522393	0.041668875	54205, 6647, 596, 1432, 581, 7124, 5868, 847, 5879, 7157, 5534
BIOCARTA	h_RacCycDPathway: Influence of Ras and Rho proteins on G1 to S Transition	10	2.872282609	0.003882954	0.049101815	595, 1021, 1869, 387, 5970, 1019, 5058, 1027, 4792, 5879

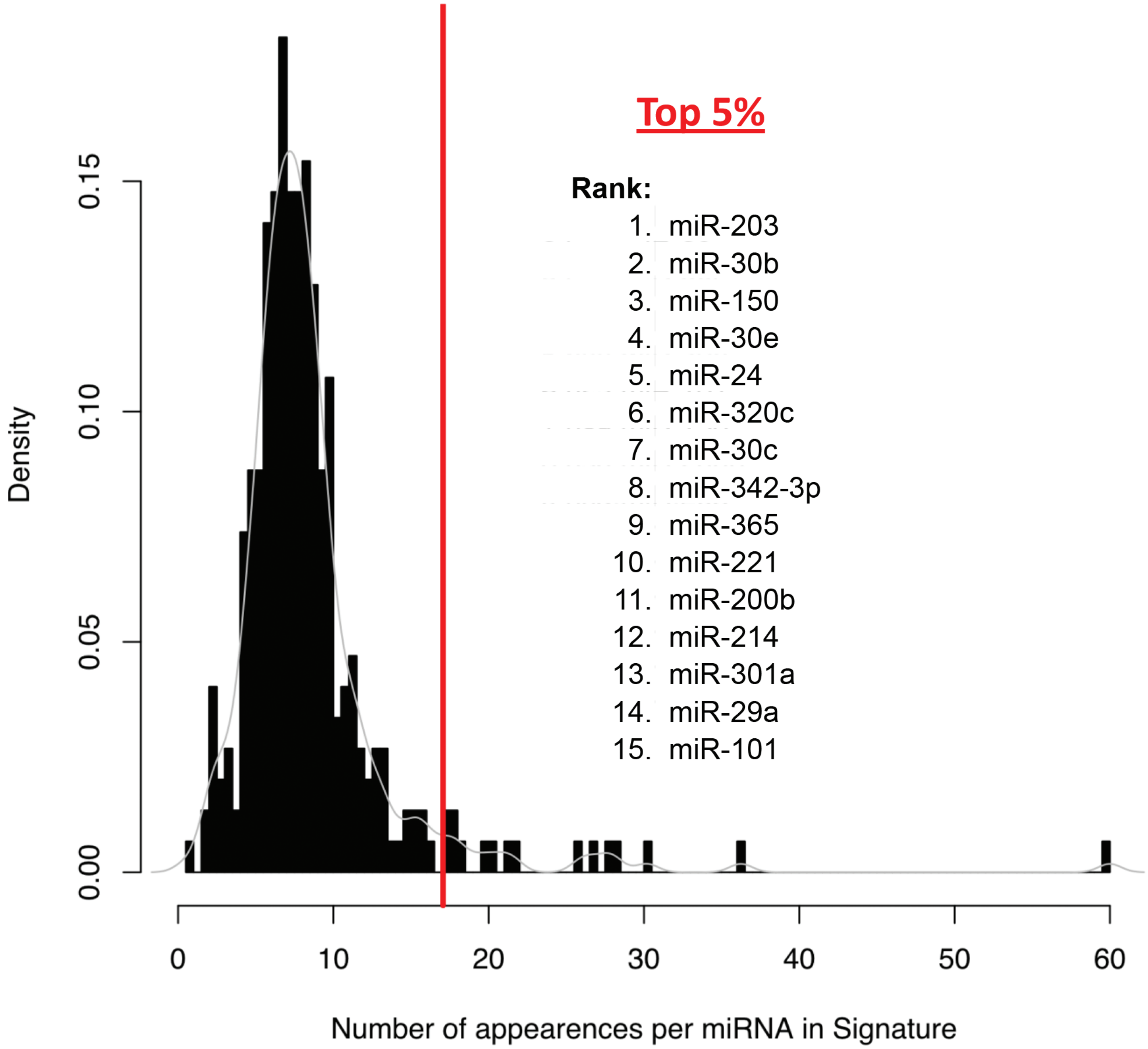

Figure S4: Distribution of the number of times each miRNA appears in a random miRNA signature with a p-value ≤ 0.014; that of the 4-miRNA signature identified herein. The 5% most frequently occurring miRNAs are listed to the right of the red line, ranked in order from most to least frequently occurring.

Supplementary Table S5: Univariate and multivariate CoxPH analysis of clinical factors and miRNA-signature Risk-Score in the Validation cohorts only (n = 121)

	Univariate		Multivariate	
	HR (95% CI)	*p*-value	HR (95% CI)	*p*-value
**MiRNA RS (High Risk *vs.* Low Risk)**	3.2 (1.2–8.5)	0.01	3.4 (1.3–9.1)	0.02
**T stage (T1&2 *vs.* T3&4)**	1.6 (0.6–4.0)	0.34	1.6 (0.6–4.2)	0.39
**N stage (N0&1 *vs.* N2&3)**	1.8 (0.7–4.6)	0.2	1.9 (0.7–4.9)	0.19
**Age**	1.0 (0.9–1.0)	0.62	1.0 (1.0–1.0)	0.56
**Gender (Female *vs.* Male)**	1.2 (0.4–3.8)	0.7	1.3 (0.4–4.1)	0.62
**Chemotherapy (− *vs.* +)**	1.0 (0.4–2.8)	0.99	0.6 (0.2–1.9)	0.43

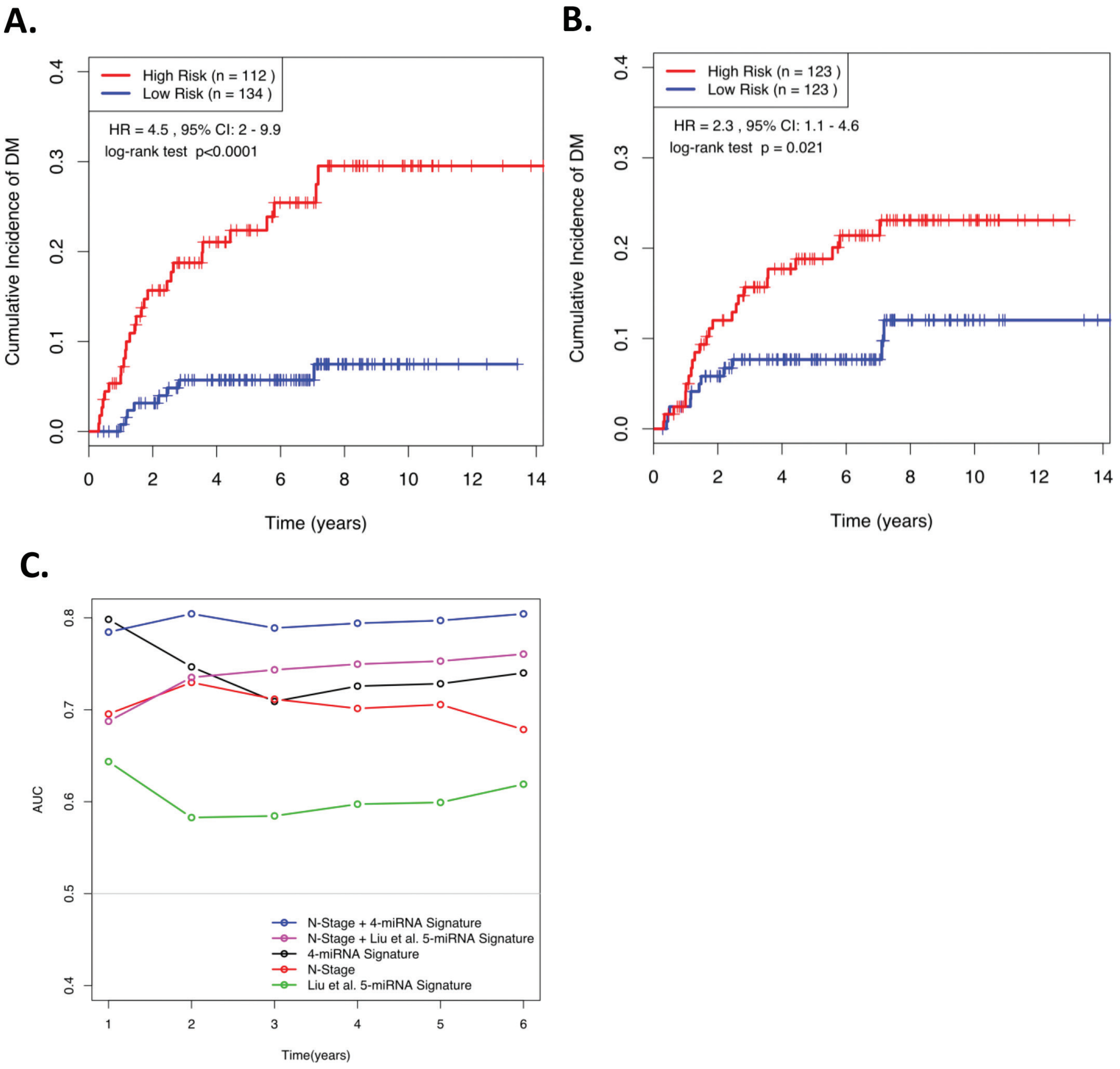

Figure S5: (A&B) Kaplan-Meier curves showing distant relapse in the combined data set including both the training and validation cohorts (n=246). Patients were dichotomized by A) the 4-miRNA signature described in the current study or B) the 5-miRNA signature described by Liu et al.[34]. “High Risk” is defined as a RS ≥ the median in the training set, and “Low Risk” is defined as a RS < the median in the training set. C) ROC AUCs across multiple time points showing the ability of the 4-miRNA signature described herein, the 5-miRNA signature reported by Liu et al.[31] and N-stage alone or combined with either signature to predict DM in NPC patients. These data demonstrate that the model including N-stage and the 4-miRNA signature is the most robust prognostic signature. HR; Hazard Ratio, CI; Confidence Interval, N; Nodal, ROC; receiver operating characteristic. AUC; Area under the curve, RS; Risk Score.

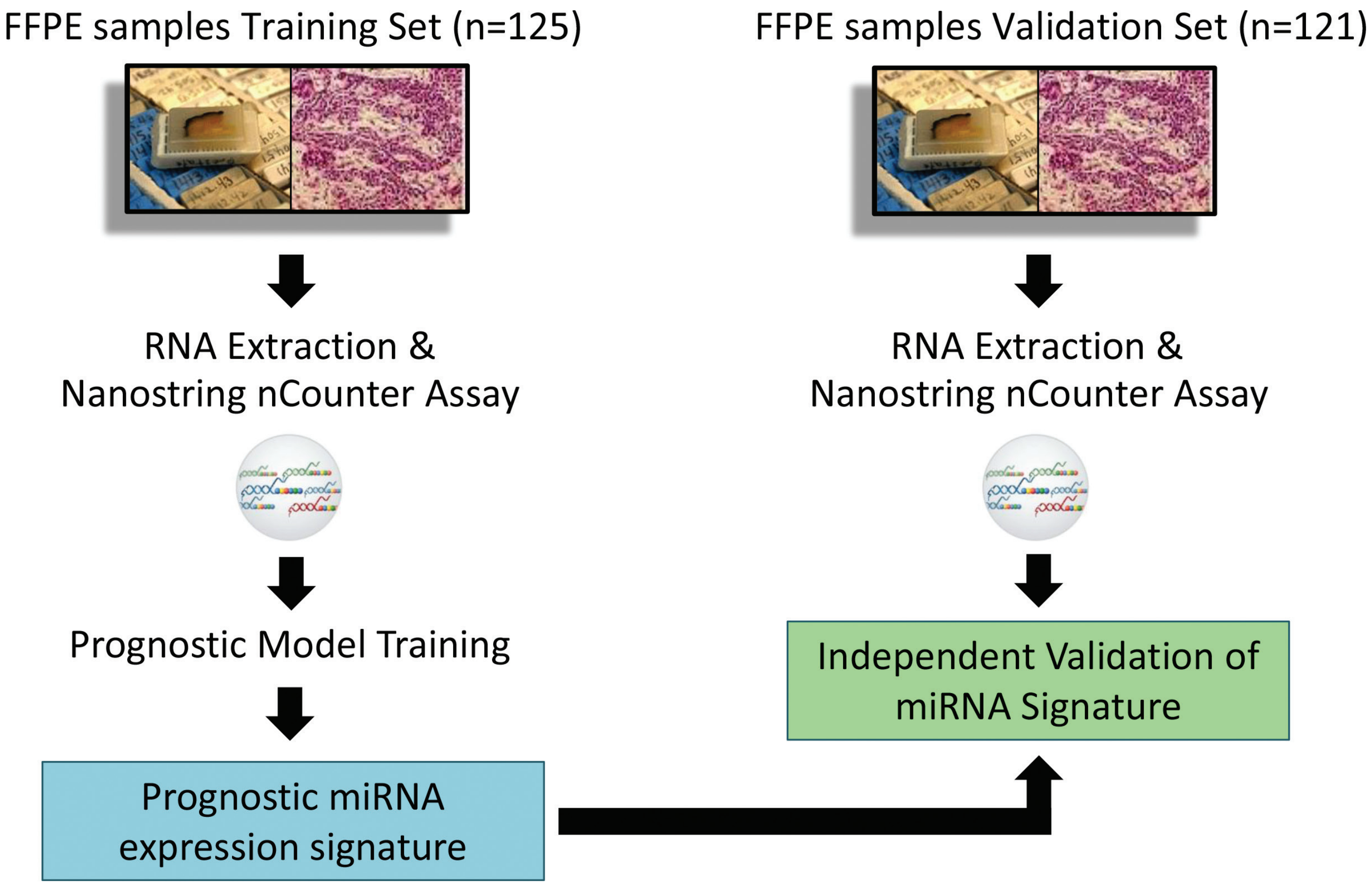

Figure S6: Schematic diagram showing miRNA signature generation and validation workflow used in the current study.
